# Supplementary material for: Seminoma and Embryonal Carcinoma Footprints Identified by Analysis of Integrated Genome-Wide Epigenetic and Expression Profiles of Germ Cell Cancer Cell Lines
Source: PLoS One. 2014 Jun 2;9(6):e98330. doi: 10.1371/journal.pone.0098330 (PMC4041891; doi:10.1371/journal.pone.0098330)
Supplement: File S1 — ZIP file containing DMRforPairs output for significant regions. Please start from the html files. (ZIP) [file pone.0098330.s008.zip › figures/35529.pdf]

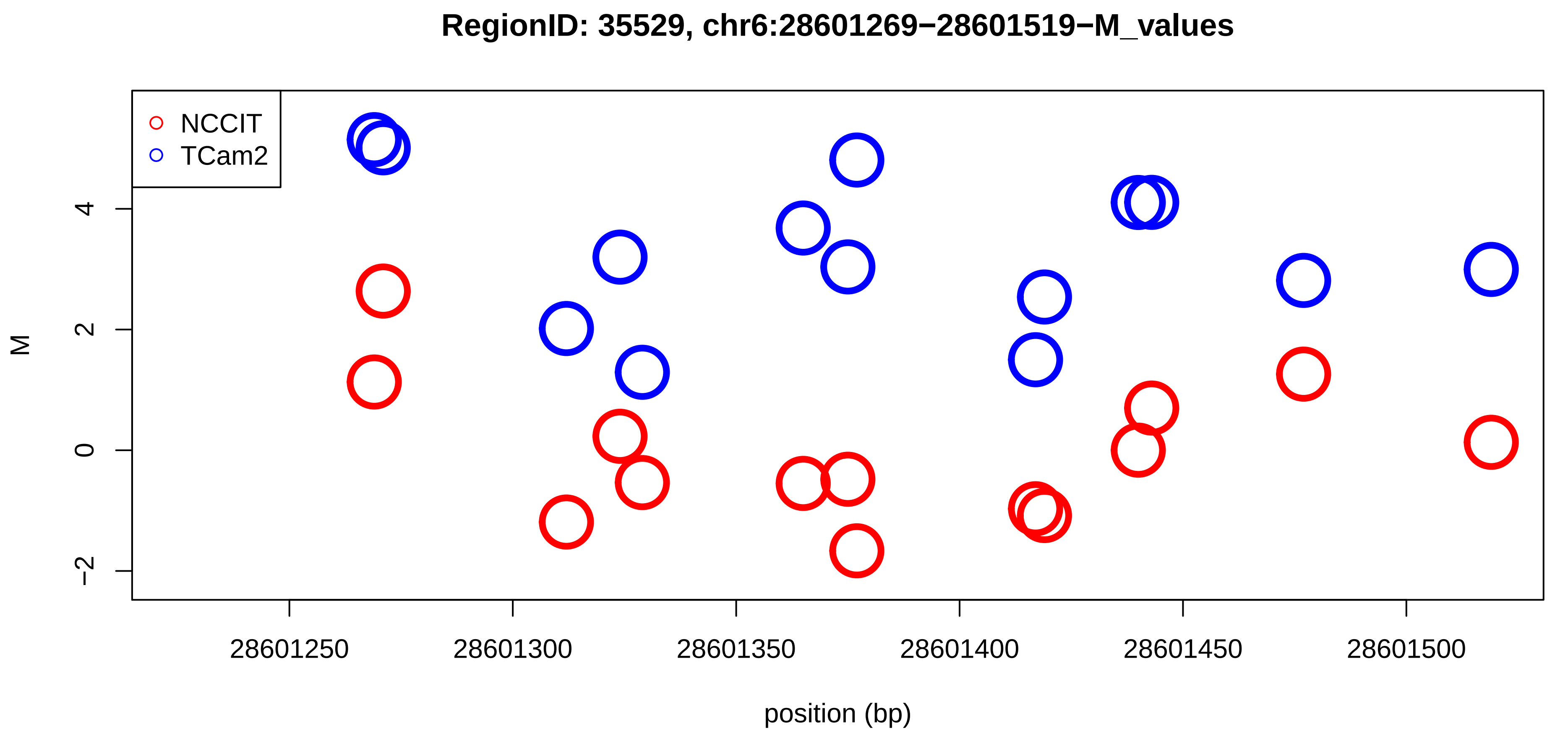
RegionID: 35529, chr6:28601269−28601519−M_values
NCCIT
TCam2
M
−2
0
2
4
28601250
28601300
28601350
28601400
28601450
28601500
position (bp)

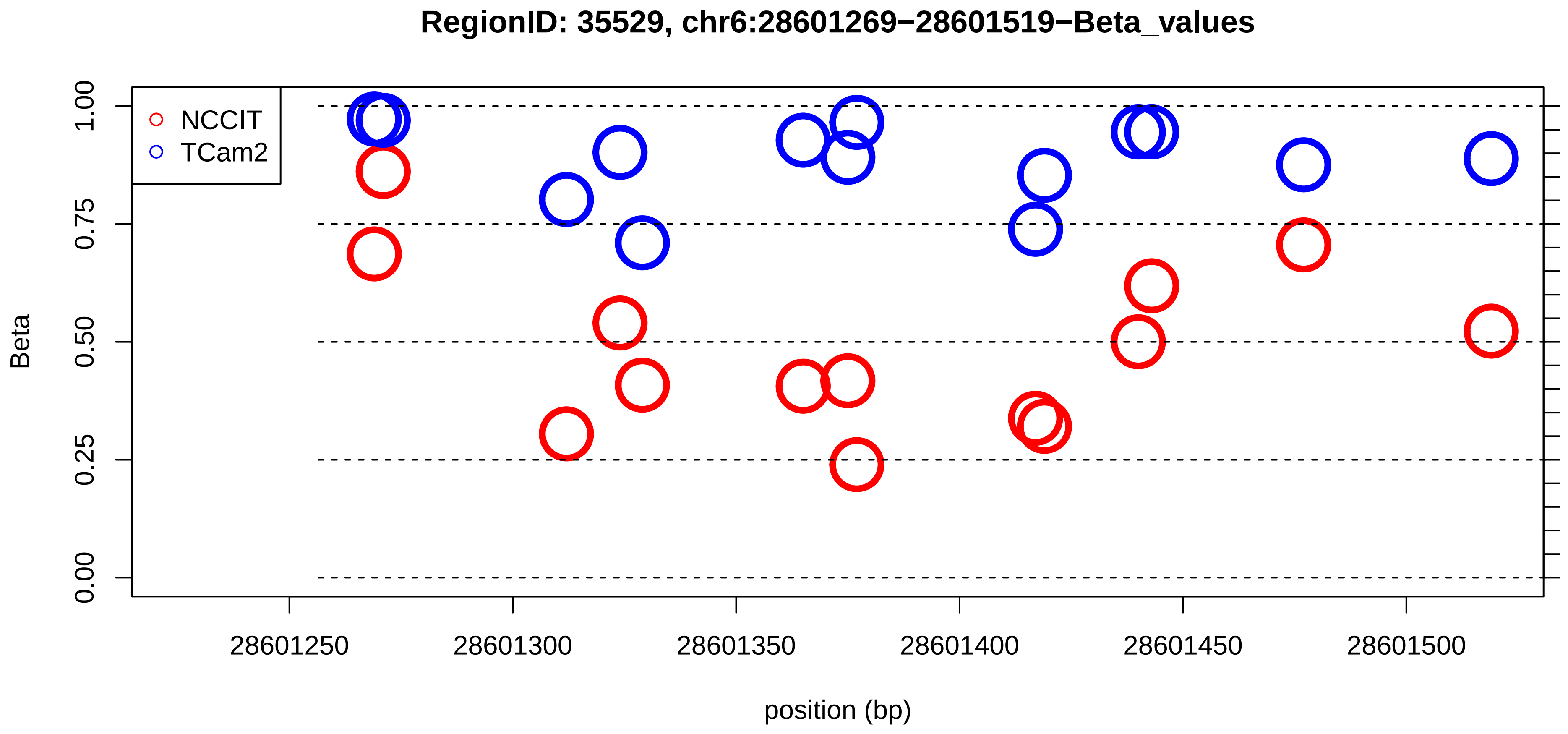
RegionID: 35529, chr6:28601269−28601519−Beta_values
NCCIT
TCam2
Beta
0.00
0.25
0.50
0.75
1.00
28601250
28601300
28601350
28601400
28601450
28601500
position (bp)

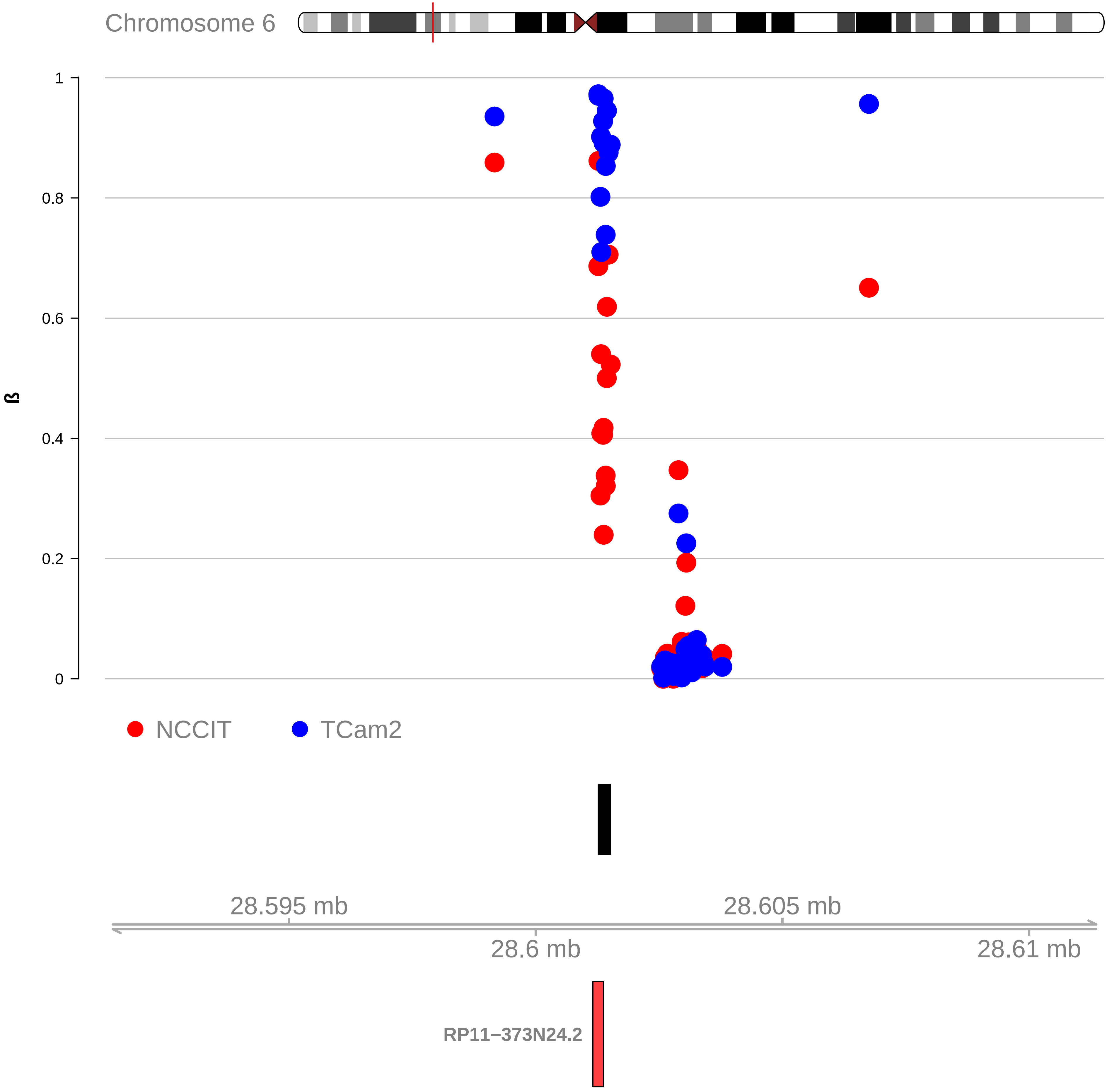

Chromosome 6
1
0.8
0.6
0.4
0.2
0
ß
NCCIT
TCam2
28.595 mb
28.605 mb
28.6 mb
28.61 mb
RP11−373N24.2
